# Self-efficacy beliefs and expectations during an Emergency Medicine Clerkship

**DOI:** 10.1186/s12245-021-00406-0

**Published:** 2022-01-22

**Authors:** Arif Alper Cevik, Elif Dilek Cakal, David Alao, Margret Elzubeir, Sami Shaban, Fikri Abu-Zidan

**Affiliations:** 1 Department of Internal Medicine, Emergency Medicine Section, College of Medicine and Health Sciences, Al-Ain, United Arab Emirates; 2 Department of Emergency Medicine, Tawam Hospital, Al-Ain, United Arab Emirates; 3 Masters of Medical Education, University of Dundee, Dundee, UK; 4 Department of Medical Education, College of Medicine and Health Sciences, United Arab Emirates University, Al-Ain, United Arab Emirates; 5 Department of Surgery, College of Medicine and Health Sciences, United Arab Emirates University, Al-Ain, United Arab Emirates

**Keywords:** Self-efficacy, Medical student, Emergency medicine, Clerkship, Educational environment

## Abstract

**Background:**

Undergraduate emergency medicine (EM) training is important because all medical graduates are expected to have basic emergency knowledge and skills regardless of their future speciality. EM clerkship should provide opportunities to improve not only knowledge and skills but also the self-efficacy of learners. This study aims to evaluate the expectations, opinions, and self-efficacy beliefs of medical students during a 4-week mandatory EM clerkship.

**Methods:**

This study used a prospective longitudinal design with quantitative and qualitative survey methods. It includes final year medical students of the 2015–2016 academic year. Voluntary de-identified pre- and post-clerkship surveys included 25 statements. The post-clerkship survey included two open-ended questions asking participants to identify the best and worst three aspects of EM clerkship. Responses were analysed to determine themes or commonalities in participant comments indicative of the EM clerkship learning experiences and environment.

**Results:**

Sixty-seven out of seventy-nine (85%) students responded to both pre- and post-clerkship surveys. Medical students’ expectations of EM clerkships’ effect on knowledge and skill acquisition were high, and a 4-week mandatory EM clerkship was able to meet their expectations. Medical students had very high expectations of EM clerkships’ educational environment. In most aspects, their experiences significantly exceeded their expectations (*p* value < 0.001). The only exception was the duration of clerkship, which was deemed insufficient both at the beginning and at the end (*p* value: 0.92). The students perceived that their self-efficacy improved significantly in the majority of basic EM skills and procedures (*p* value < 0.001). Emergent qualitative themes in the study also supported these results.

**Conclusion:**

This study showed that a 4-week mandatory EM clerkship increased medical students' perceived self-efficacy in basic emergency management skills. The EM clerkship met students' expectations on knowledge and skill acquisition, and exceeded students’ expectations on educational environment.

## Introduction

Undergraduate emergency medicine (EM) training is important because all medical graduates, regardless of their future speciality, are likely to have to deal with unexpected emergencies at some point in their careers. In addition to their professional duties in the hospital, medical graduates in many countries have an ethical obligation to provide medical care if they encounter out-of-hospital emergencies [1, 2]. Furthermore, in some contexts, medical graduates practice as independent acute care physicians without further residency training [3]. Therefore, at the end of their undergraduate training, medical students must be knowledgeable and confident about basic acute care management.

To improve medical students’ acute care management skills and confidence, EM clerkships, where medical students are exposed to unique acute care encounters, are essential components of medical school curricula [4, 5]. However, currently, there is a lack of consensus on ideal EM clerkship design, including the duration and content [6–11]. To be able to adjust EM clerkships according to learners’ needs, we must measure its effects on trainees. The majority of published works have focused on measuring knowledge and skill acquisition, with very few addressing the issues of educational environment expectations and confidence of medical students on certain aspects of emergency care. Therefore, more studies regarding trainees’ educational environment expectations and confidence are required to create optimal clerkships.

Self-efficacy, a central concept of social cognitive theory, refers to an individual’s self-belief or self-confidence in performing a specific task to attain a certain standard [12, 13]. The level of self-efficacy determines the goals that the learner chooses, the level of commitment to learning, willingness to face challenges, resilience, and achievements [12]. Therefore, training should provide opportunities to improve not only knowledge and skills but also the self-efficacy of learners. Self-efficacy can be improved by previous successful attempts and witnessing role models’ or peers’ successful performances in a safe and encouraging learning environment [14]. A clinical clerkship should improve trainees’ self-efficacy in addition to knowledge and skills to be considered effective.

EM clerkships can provide learning opportunities in various learning environments ranging from classroom-based learning to authentic clinical experience, which are necessary tools for increased self-efficacy. However, the interplay among multiple influences on self-efficacy, including intrapersonal influences and environmental forces, affects self-effıcacy development [15, 16]. Vicarious experiences such as observing someone similar to oneself succeed at a task in a particular learning environment can increase self-efficacy [17]. Negative expectations of a learning environment may intensify anxiety and stress, while positive experiences and expectations may heighten motivation and self-efficacy beliefs. Closely related to self-efficacy beliefs are expectancy-value and expectancy theory of motivation [18, 19], which posits that individuals’ actions are related to their expectations, valuations, perceptions, and desire for continued growth, explaining how motivation is influenced by the expectancy of how well individuals will perform a task or activity. In this sense, students’ expectations and opinions about the EM clerkship are as relevant to self-efficacy as vicarious and hands-on learning opportunities provided by the clerkship.

This study aims to evaluate the expectations, opinions and self-efficacy beliefs of medical students during a 4-week mandatory EM clerkship and has two research questions: (1) to what extent can an EM clerkship meet students’ expectations regarding knowledge and skill acquisition, and educational environment? and (2) to what extent can an EM clerkship increase medical students' self-efficacy in basic emergency skills?

## Materials and methods

### Ethical approval

This study is approved by The United Arab Emirates University (UAEU) Research and Graduate Studies Ethics Committee (ERS_2017_5496).

### Study setting and participants

This study was based on the EM clerkship experience of the 2015–2016 final year medical students of the College of Medicine and Health Sciences, UAEU, Al Ain, United Arab Emirates [20].

The medical education department establishes the student groups at the beginning of each academic year. For the 2015–2016 academic year, there were three female and two male student groups. Each group has 12 to 19 students. The groups rotate through the five senior clinical clerkships of EM (4 weeks), family medicine (4 weeks), pediatrics (8 weeks), internal medicine (8 weeks), and surgery (8 weeks).

### EM clerkship design

Our EM clerkship is 4 weeks long and is undertaken during the final (sixth) year of medical school. The clerkship’s teaching and learning activities are designed according to international undergraduate curriculum recommendations [6, 21]. In addition to teaching sessions in the classroom and simulation centre, students participate in clinical shifts under the supervision of residency-trained emergency physicians and residents in two medical school-affiliated community teaching hospitals. In total, the emergency departments of both hospitals treat over 200,000 patients annually (Tawam Hospital ED treats approximately 105,000–110,000 patients, and Al Ain Hospital ED treats approximately 110,000–115,000 patients annually). Sundays and Thursdays are reserved for classroom and simulation-based learning respectively while the other days of the week are allocated for clinical shifts. The clerkship includes various formative and summative assessments such as work-based assessments, written tests, Objective Structured Clinical Examination (OSCE), and logbooks for patient and procedure encounters. The details of the EM clerkship curriculum and students’ logbooks can be found elsewhere [22, 23].

### Study design and data collection

This study used prospective longitudinal design with quantitative and qualitative survey methods. A previously published survey [10] was adapted to our context. A voluntary de-identified survey was taken on the first day of clerkship (pre-clerkship survey) and the last day of the clerkship (post-clerkship survey). The survey included 25 statements to capture students’ pre-clerkship expectations and post-clerkship opinions regarding knowledge and skill acquisition, educational environment, and self-efficacy. Each statement was evaluated using a five-point Likert Scale, from strongly disagree to strongly agree. In addition, a post-clerkship survey included two open-ended questions asking participants to identify the three best and worst aspects of EM clerkship. We did not add any new material or method to the curriculum, and no modifications were made prior to the beginning of the study to impact the students’ responses. Responses were analysed to determine themes or commonalities in participant comments indicative of the EM clerkship learning experiences and environment. Each student used a matching code in both surveys to protect students’ privacy and avoid the influence of power differentials. The online survey/assessment tool Socrative [24] was used to conduct the surveys. The results were extracted into an excel sheet, cleaned, and prepared for the data analysis.

### Data analysis

To compare students’ responses between the pre-clerkship and post-clerkship surveys, the non-parametric Wilcoxon signed-rank test was used. The internal consistency reliability of the survey was calculated using Cronbach’s alpha coefficient. To measure the strength and direction of the association between two variables, Spearman’s rank correlation was used. The Statistical Package for the Social Sciences (IBM-SPSS version 26, Chicago, IL) was used for quantitative analysis. Statistical significance is accepted at a level less than 0.05 of *p* value. Two of the authors manually analysed the qualitative data for congruence and faithful representation of emerging themes..

## Results

Seventy-nine final year medical students attended a 4-week mandatory EM clerkship during the study period of which 54 (68%) were female and 25 (32%) were male. The number of students in each of the five groups ranged from 12 to 19. Sixty-seven students (85%) responded to both pre-clerkship and post-clerkship surveys and were included in the analysis. A Cronbach’s alpha of 0.89 was observed for the pre-clerkship survey, indicating good internal consistency of this survey.

Table 1 shows pre-clerkship scores of expectations and post-clerkship scores of opinions. Regarding perceived knowledge and skill acquisition, the means of all pre-clerkship scores were above 4 (agree), which shows high expectations from the clerkship at the beginning. Post-clerkship mean scores did not change significantly (*Wilcoxon signed ranks test*, *p* value: 0.33). Students’ scores on the educational environment of EM clerkship improved significantly (*Wilcoxon signed ranks test*, *p* value < 0.001). However, students’ scores on the appropriateness of the EM clerkship duration did not change after the clerkship (*Wilcoxon signed ranks test*, *p* value: 0.92).

**Table 1:** Pre-clerkship scores of expectations and post-clerkship scores of opinions

	Pre-clerkship scores		Post-clerkship scores		
Category and questions	Median (min–max)	Mean (SD)	Median (min–max)	Mean (SD)	***p*** value
**Knowledge and skill acquisition** ***I believe, in the EM clerkship, I wilI/did***	4.50 (3–5)	4.38 (0.56)	4.25 (2–5)	4.32 (0.57)	0.329
Increase my knowledge	5 (1–5)	4.60 (0.82)	5 (2–5)	4.43 (0.66)	0.057
Gain self-confidence to manage emergency cases	4 (2–5)	4.31 (0.70)	4 (2–5)	4.28 (0.67)	0.664
Gain self-confidence to do basic emergency procedures	4 (2–5)	4.22 (0.74)	4 (2–5)	4.21 (0.69)	0.907
Encounter a variety of patients	4 (2–5)	4.37 (0.71)	4 (2–5)	4.37 (0.71)	0.985
**Educational environment**	3.6 (2.6–4.9)	3.57 (0.41)	4 (2.9–5)	3.93 (0.44)	< 0.001
***I believe,***					
EM clerkship duration (4 weeks) is appropriate	2(1–5)	2.43(1.03)	2(1–5)	2.46(1.34)	0.917
Clerkship director is easily reachable	4(3–5)	4.04(0.71)	5(3–5)	4.57(0.61)	<0.001
EM faculty members are easily reachable	4(2–5)	3.88(0.75)	4(1–5)	4.25(0.84)	0.006
Grading percentages and process are appropriate	3(2–5)	3.55(0.66)	4(3–5)	3.96(0.75)	0.001
EM clerkship is overall good learning experience	4(3–5)	4.03(0.63)	4(2–5)	4.40(0.68)	<0.001
I consider choosing EM as an additional elective rotation	4(1–5)	3.48(1.06)	4(1–5)	3.96(0.98)	0.002
I prefer to take additional EM course in early years	4(1–5)	3.58(1.03)	4(2–5)	3.94(1.03)	0.022

*p* value Wilcoxon signed ranks test

Table 2 shows pre- and post-clerkship scores of perceived self-efficacy in specific patient management and procedural skills. The overall change was significant (*Wilcoxon Signed Ranks test*, *p* value < 0.001). After the clerkship, students’ scores improved significantly in eight out of 13 statements. The scores for formulating a differential diagnosis list, developing an appropriate management plan, inserting a Foley catheter and inserting IV cannula/venipuncture were improved but did not show a significant change.

**Table 2:** Pre- and post-clerkship scores of perceived self-efficacy

	Pre-clerkship scores		Post-clerkship scores		
Category and questions	Median (min–max)	Mean (SD)	Median (min–max)	Mean (SD)	***p*** value
***I am able to***					
Conduct an initial assessment of an acutely ill patient	4(2–5)	4.04(0.78)	4(3–5)	4.25(0.59)	0.045
Identify a “sick” vs. “non-sick” patient	4(2–5)	3.97(0.74)	4(3–5)	4.25(0.53)	0.013
Formulate a differential diagnosis list	4(3–5)	4.21(0.66)	4(2–5)	4.22(0.62)	0.861
Develop an appropriate management plan	4(3–5)	3.97(0.67)	4(2–5)	4.07(0.61)	0.312
Present cases in a formal setting	4(2–5)	3.37(0.94)	4(3–5)	3.73(0.86)	0.019
Perform wound closure and repair	4(1–5)	3.58(0.92)	4(2–5)	4.04(0.75)	0.003
Insert Foley catheter	4(1–5)	3.51(0.96)	4(1–5)	3.60(0.94)	0.511
Interpret EKG	4(1–5)	3.97(0.80)	4(3–5)	4.30(0.70)	0.005
Perform basic life support	4(2–5)	3.76(0.87)	4(3–5)	4.30(0.60)	< 0.001
Participate medical resuscitation	3(1–5)	3.21(1.01)	4(3–5)	4.10(0.68)	< 0.001
Participate trauma resuscitation	3(1–5)	3.15(0.97)	4(3–5)	4.21(0.66)	< 0.001
Perform splinting/fracture care	3(2–5)	3.31(1.02)	4(1–5)	3.85(0.88)	< 0.001
Place an IV access/venipuncture	4(3–5)	4.07(0.75)	4(3–5)	4.27(0.67)	0.121
Place a NG tube	4(2–5)	3.78(0.85)	4(1–5)	3.51(0.91)	0.058
**Overall**	3.71 (2.3–5)	3.75 (0.58)	4 (2.9–5)	4.08 (0.48)	< 0.001

*p* value Wilcoxon signed ranks test

The students’ pre-clerkship scores of perceived knowledge and skill acquisition were moderately correlated with pre-clerkship scores of perceived self-efficacy (*Spearman’s Rho* 0.53, *p* value < 0.001). Pre-clerkship scores of educational environment showed a weak correlation with pre-clerkship scores of perceived self-efficacy (*Spearman’s rho* 0.27, *p* value 0.03).

The students’ post-clerkship scores of perceived knowledge and skill acquisition showed a strong correlation with post-clerkship scores of perceived self-efficacy (*Spearman’s rho* 0.70, *p* value < 0.001). Post-clerkship scores of educational environment showed moderate correlation with students’ post-clerkship scores of perceived self-efficacy (*Spearman’s rho* 0.49, *p* value < 0.001).

The difference between post- and pre-clerkship scores of knowledge and skills acquisition were moderately correlated with the difference between post- and pre-clerkship scores of perceived self-efficacy (*Spearman’s rho* 0.47, *p* value < 0.001). The difference between post- and pre-clerkship scores of educational environment were moderately correlated with difference between post- and pre-clerkship scores on perceived self-efficacy (*Spearman’s rho* 0.41, *p* value < 0.001).

In qualitative analysis, emerging themes about positive aspects of EM clerkship included numerous and diverse opportunities for hands-on training including patient encounters and procedures; helpful and supportive program director, and healthcare staff including attending physicians, residents and nurses; an organised and well-structured clerkship; high quality and diversity of teaching methods and materials. Additionally, the students stated that EM clerkship increased knowledge and confidence in patient management and procedures. Their main complaint was the short duration of EM clerkship. In addition, sleep deprivation due to shifts, difficulty filling logbook due to computer-based systems, and the suboptimal attitude of some healthcare staff were emerging themes. Also, students stated that they wished to receive additional EM training in the early years of medical school.

## Discussion

Our results showed that medical students’ expectations on knowledge and skill acquisition were high and a 4-week mandatory EM clerkship was able to meet expectations. Similarly, they had very high expectations on educational environment. In most aspects of the educational environment, their experience exceeded their expectation significantly. The only exception was the duration of clerkship, which was deemed insufficient both at the beginning and at the end. The students perceived that their self-efficacy in the majority of basic skills and procedures improved significantly. These results were also supported by emergent qualitative themes in the study.

Medical students’ expectations of EM clerkships are affected by many factors. EM’s popularity by mainstream and social media, students’ previous exposure to emergency departments, and previous students’ positive experiences with EM clerkship may affect students' expectations [25–27]. In our setting, students’ expectations of EM clerkship regarding knowledge and skill acquisition were quite high. The high expectation from EM clerkships has been well documented in the literature [27]. Because of such high expectations, it may not always be possible to observe a significant increase in scores after the clerkship. Even if the post-clerkship scores were not increased in our study, they remained stable, which may show that the EM clerkship met students’ expectations. Inclusion of students’ expectations of learning environment provides insight into a potential building block to students’ self-efficacy. Perceptions of a structured, supportive learning environment, involvement in a variety of patient encounters and procedures and role modelling by multidisciplinary team members, observed in our study, enhanced positive expectations of the utility of the learning experience which in turn supported self-efficacy.

The implementation of EM clerkships in medical school curricula vary significantly. In the United States, a pioneer in EM training, the percentage of medical schools implementing mandatory EM clerkships was only 52% as of 2014 [28]. In addition, when to implement EM clerkship in medical school curriculum is controversial [28, 29]. Despite the suggested ideal duration of at least 4 weeks, the duration of EM clerkships varies among countries, ranging from 1 to 6 weeks [7, 30–32]. Even in a mature system, at least 18% of EM clerkships remained shorter than 4 weeks [28]. We have implemented a 4-week mandatory EM clerkship in the final year of a 6-year medical school program since 2013 [22]. Though our students’ perceived self-efficacy was increased significantly during 4 weeks and the duration of our EM clerkship is aligned with the recommendations, our results showed that students deemed 4 weeks of emergency medicine clerkship too short. Also, they seem to prefer taking additional EM training in earlier years.

EM clerkship provides an excellent opportunity for medical students to practice fundamental topics and procedures and facilitates knowledge acquisition and self-efficacy in basic procedures and patient management skills [33–35]. Similar to reports by Avegno et al. [10] our medical students stated high self-efficacy values at the beginning of the EM clerkship with significantly increased values on 8 of 13 items. The items with the greatest perceived self-efficacy change were the items that are mostly exclusive to emergency departments, such as basic life support, medical, and trauma resuscitations.

There are several ways to boost the self-efficacy of a learner. Previous successes, performed by self or observations of others, raise self-efficacy, while failures lower it [14]. In our EM clerkship, medical students have opportunities for supervised skills and procedures, which are tailored to their levels, and opportunities for observing peers' performances [22, 23]. A supportive educational environment is also essential for self-efficacy and effective learning [36, 37]. In our study, the medical students' pre-clerkship scores of elements measuring the educational environment were high at the beginning and significantly increased at the end. Similar to previous reports, our students valued the variety of cases seen, opportunities to develop clinical skills, teachers’ support and commitment to learning, close supervision, and the ability to reach teachers when needed [38–40]. We note that their positive perception of the educational environment may have contributed to increasing self-efficacy, as ultimately, the greater the expectations of the learning environment are met, the greater the potential for a perceived sense of control and self-efficacy.

Self-efficacy is associated with motivation, which greatly facilitates learning. In undergraduate medical education, a higher level of motivation increases academic success and performance [41]. The motivational dynamic model suggests that self-efficacy is a key determinant of motivation to learn [42]. Therefore, medical teachers should promote motivation during teaching and learning activities. In our study, students’ increased interest in additional EM training suggests that their motivation to learn EM had increased with their self-efficacy.

In addition to its relation to motivation and expectancy beliefs, self-efficacy has a sophisticated relation to performance [43]. Mavis [44] examined the relationship between several constructs, including self-efficacy and OSCE score. He found that although there was no overall correlation between self-efficacy and OSCE score, the students with high self-efficacy performed significantly better at OSCE than students with low self-efficacy. Based on the correlations between constructs, he created a framework which suggests that an increase in biomedical knowledge and clinical skills improve self-efficacy by decreasing anxiety, and self-efficacy promotes performance by increasing preparedness [44]. Self-efficacy seems to contribute to performance but not exclusively; personal attributes also apply [45]. Conversely, self-efficacy not rooted in reality might hinder performance because it makes novice learners more prone to errors [46]. In our study, we compared self-efficacy with students’ perceptions of knowledge and skill acquisition rather than objective assessment scores. Students’ perceptions of knowledge and skill acquisition seemed correlated with self-efficacy; however, students’ self-evaluated performance may not always reflect objective assessment scores. Also, the correlation post-clerkship self-efficacy and post-clerkship perceptions on learning environment may be due to overlap among these constructs. Bandura [15, 16] also included expectancies in his discussion of self-efficacy development. Understanding effect sizes of related constructs could be an important contribution to the study of these variables in medical education [43]. Recognising the impact of perceptions and expectations of the learning environment on self-efficacy beliefs could be helpful in the development of EM clerkship curricula, and identif and support students at risk. Educators may increase knowledge acquisition by promoting self-efficacy. Self-efficacy boosts motivation, and motivation may lead to knowledge increase, creating a beneficial cycle.

## Limitations

There are some limitations in our study. *First*, this was a single-center study conducted over one academic year having a small sample size. *Second*, there was no control group to compare the effect of mandatory versus elective clerkships, different clerkship durations and different EM clerkship years. *Third*, we did not use a previously validated self-efficacy scale, which may provide more structured and objective information [47, 48]. Cognizant that there is no single all-purpose measure of self-efficacy, since the construct is not a generalised trait and people differ in efficacy across different domains, we constructed a scale based on sound conceptual determinants of self-efficacy in an undergraduate task-related EM domain. *Fourth*, we did not compare OSCE and written assessment scores with students' self-efficacy belief scores, which could give additional information regarding students' actual gain in the clerkship. Instead, we measured subjective expectations and opinions on knowledge and skills acquisition, and educational environment. *Fifth*, the clerkship is modelled after international undergraduate curriculum recommendations, which may differ from the curricula used at other institutions, limiting the generalisability of the study. *Finally*, we acknowledge there is scope for longitudinal research, evaluating a broader set of related constructs and changes in self-efficacy.

## Conclusions

This study has shown that a 4-week mandatory EM clerkship increased medical students' perceived self-efficacy in basic emergency management and skills. It also met students’ expectations on knowledge and skill acquisition and exceeded students' expectations on the educational environment. Considering their contributions to learning, motivation, and performance, self-efficacy and expectations of educational environment and activities are valuable factors in developing and measuring EM clerkships. Future studies could focus on how changes to the curricula could affect self-efficacy beliefs and could also look at whether there is a correlation between self-efficacy beliefs and performance by comparing the self-efficacy scores of students to OSCE, work-based assessments, or written test scores.
